# Navigating the Complexity of Bilingual Aphasia: Current Insights and Future Directions

**DOI:** 10.3390/brainsci15090989

**Published:** 2025-09-14

**Authors:** Marissa Russell-Meill, Manuel J. Marte, Erin Carpenter, Swathi Kiran

**Affiliations:** Center for Brain Recovery, Department of Speech, Language, and Hearing Sciences, Boston University, 111 Cummington Mall, Boston, MA 02215, USA; mruss@bu.edu (M.R.-M.); mjmarte@bu.edu (M.J.M.); evc5102@bu.edu (E.C.)

**Keywords:** bilingual aphasia, aphasia recovery, cross-language generalization, machine learning, stroke rehabilitation, neuroimaging, neuromodulation, treatment outcomes

## Abstract

Bilingual aphasia is shaped by a dynamic interplay of neural, cognitive, linguistic, and experiential factors that influence both impairment and recovery. This review synthesizes current evidence on bilingual language organization, assessment, and treatment, emphasizing how individual language histories and cognitive systems contribute to variability in outcomes. We highlight the challenges of estimating pre-stroke proficiency, evaluating impairment across languages, and interpreting recovery patterns. Finally, we explore emerging technological directions while emphasizing that advances in machine learning, automated assessment, and neurotechnology must be developed with explicit attention to cultural responsiveness and equity to ensure benefits reach diverse multilingual populations.

## 1. Introduction

Aphasia, an acquired language disorder most commonly caused by stroke, affects approximately one-third of stroke survivors and remains one of the most significant barriers to post-stroke recovery and quality of life [1,2,3,4]. While the study of bilingual aphasia has advanced in recent years, foundational research and clinical practice continue to reflect a monolingual orientation. This stands in stark contrast to global linguistic realities: over half the world’s population speaks more than one language, and rates of bilingualism are steadily increasing [5]. As a result, clinicians treating bilinguals with aphasia (BWA) frequently rely on tools, frameworks, and evidence developed for monolingual populations.

Bilingual aphasia introduces theoretical and clinical challenges that differ fundamentally from monolingual cases. The interaction of multiple languages within the bilingual brain gives rise to distinct patterns of impairment and recovery—patterns that often fall outside the predictions of monolingual frameworks [6,7,8,9,10,11,12]. These differences span neurobiological mechanisms, clinical presentations, assessment methods, and treatment outcomes, and are increasingly understood through studies on how bilingual experience shapes language-relevant brain networks [13,14]. As such, they necessitate specialized knowledge and interventions tailored to bilingual populations.

**Figure 1:**
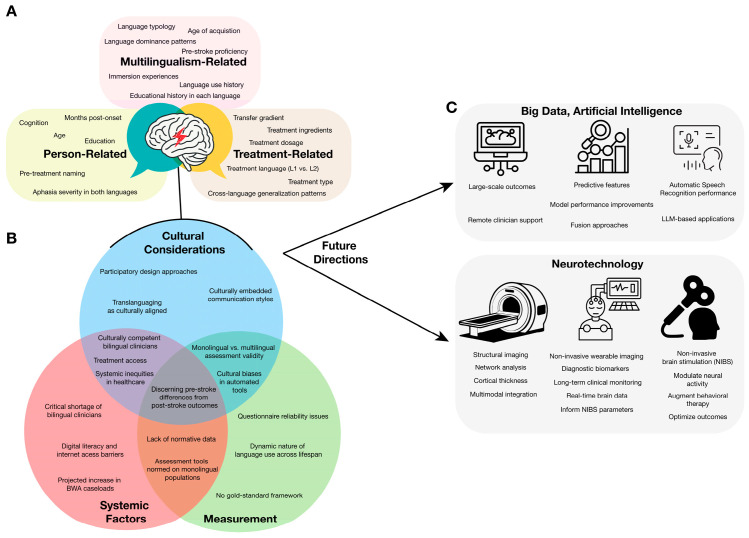
Bilingual aphasia: from current challenges to future directions. This conceptual framework illustrates the multifaceted nature of bilingual aphasia research and clinical practice. (**A**) Influential factors: key variables that shape assessment and treatment outcomes, including multilingualism-related (e.g., [16,17,18,19]), person-related (e.g., [20,21,22,23]), and treatment-related (e.g., [24,25,26,27,28]) variables. (**B**) Current challenges: major barriers facing bilingual aphasia care, encompassing systemic issues (e.g., [2,5,29,30], measurement limitations (e.g., [31,32,33,34,35]), and cultural considerations (e.g., [36,37,38,39]), with overlapping areas representing complex interactions between domains. (**C**) Future technological solutions: emerging approaches to advance rehabilitation through artificial intelligence and big data applications (e.g., [20,40,41,42,43]) and innovative neurotechnology platforms (e.g., [44,45,46,47]), offering scalable solutions for assessment, treatment delivery, and neural monitoring in diverse multilingual populations.

Efforts to address these challenges have catalyzed major advances in understanding bilingual language use and recovery. In parallel, technological innovations in machine learning, artificial intelligence, and neurotechnologies have opened new avenues for more sophisticated and culturally responsive clinical approaches. The present review synthesizes this rapidly expanding body of evidence and identifies key opportunities emerging from the convergence of research and technological innovation in bilingual aphasia (Figure 1). In doing so, we adopt an experience-based definition of “bilingualism” as the use of two languages by an individual, characterized by graded variation in factors such as age of acquisition, proficiency, exposure, and daily use (e.g., [15]). We use the term “multilingual” to describe individuals who use more than two languages, explicitly noting when evidence is derived from multilingual participants or when claims extend beyond bilingual contexts. We begin by examining current evidence regarding bilingual language organization and emerging theoretical consensus, then address the critical challenges in assessment and the development of bilingual-specific tools. We review established treatment approaches and emerging evidence on cross-language generalization before exploring future directions in rehabilitation, including machine learning applications, portable neuroimaging technologies, and naturalistic assessment paradigms. Finally, we examine cultural considerations and equity issues that must be integrated into bilingual aphasia care to ensure that scientific advances translate into accessible and effective treatment for diverse multilingual populations.

## 2. Bilingual Aphasia: What We Know and What Remains Unclear

### 2.1. Foundational Questions and Emerging Consensus

The field of bilingual aphasia has achieved consensus on several fundamental questions that guide clinical practice and theory development. Three critical questions have shaped our understanding: (1) How are multiple languages organized within the bilingual brain? (2) What mechanisms control language selection and switching? (3) How do individual factors influence the balance between shared and distinct neural representations? We review the evidence addressing each question to provide a foundation for understanding assessment and treatment approaches discussed in subsequent sections.

### 2.2. Neural Organization of Multiple Languages

#### 2.2.1. Shared Substrates with Distinct Functional Patterns

How do first and second languages organize themselves within the neural and cognitive architecture of the human brain? Bilingual aphasia provides an answer to whether bilingual lexicons are unified systems sharing common substrates or segregated networks with distinct representations, as patterns of language breakdown and recovery reveal the underlying organizational principles. Indeed, longstanding clinical observations ranging from parallel impairment across languages to selective preservation of one language system have yielded crucial insights [11,48]. After decades of systematic investigation, the evidence converges on a hybrid architecture where languages use both shared neural resources and functionally distinct control mechanisms, with the balance determined by linguistic level and individual factors.

This hybrid architecture reveals itself through multiple converging methodologies that have refined our understanding over decades. For example, intraoperative cortical stimulation studies demonstrate extensive overlap in language localization, with the same cortical sites typically supporting both languages in bilingual patients [49,50,51]. Modern neuroimaging extends these observations, revealing that apparent language-specific activation patterns often reflect differences in proficiency, usage frequency, and cognitive control demands rather than fundamental architectural separation [52,53]. Meta-analytic evidence has further clarified these patterns: BWA who shift toward L2 dominance show increased left frontal and anterior cingulate activation when processing their weaker L1, indicating that reduced usage leads to more distributed neural recruitment [54]. Similarly, healthy bilinguals demonstrate broader L2 than L1 activation, particularly in late learners, with L2 processing recruiting additional executive control regions [14,55]. Systematic differences in neural engagement modulated by dominance patterns and acquisition timing support the shared substrate hypothesis while explaining individual variability.

#### 2.2.2. Language Control and Selection Mechanisms

But if languages share anatomical substrates, how do bilinguals maintain functional separation between their linguistic systems? Population encoding theory addresses this theoretical gap by proposing that neural representations are defined by patterns of activity distributed across neural *populations* and not entirely by location [56,57]. In this framework, the same cortical regions can support multiple functions (i.e., distinct linguistic processing routines) through distinct patterns of neural firing, much as the visual cortex represents different objects through overlapping but differentiable activation patterns [58]. Applied to bilingualism, this means languages are distinguished not by occupying separate brain regions but by evoking unique distributed activation signatures within shared neural networks. In fact, recent computational and fMRI-based modeling work demonstrates how self-organizing principles can generate these language-specific representations within common anatomical substrates, providing mechanistic accounts of how languages “tune” shared conceptual representations and coexist without constant interference [19,59].

Furthermore, behavioral evidence from language switching paradigms highlights the control mechanisms operating within this shared architecture. Traditional models invoked the concept of active inhibition to prevent non-target language intrusions [60,61], whereas contemporary frameworks emphasize selection through competitive activation, where contextual and usage-based factors naturally elevate target representations [62,63]. Studies of voluntary switching in bilingual aphasia provide crucial support for this view: patients maintain a strategic language selection approach despite executive control deficits, suggesting that language choice may rely on implicit activation dynamics rather than effortful suppression [64,65,66].

#### 2.2.3. Individual Factors and Clinical Implications

The convergence of evidence from cortical stimulation, neuroimaging meta-analyses, and behavioral studies in bilingual aphasia strongly suggests that languages operate through shared neural substrates with distinct functional patterns. A hybrid architecture where individual factors (e.g., age of acquisition and usage patterns) modulate neural overlap largely explains the heterogeneous recovery patterns observed in bilingual aphasia. Critically, if languages share core representations while maintaining functional separation through distributed activation patterns [19,56,59], then accurately assessing post-stroke deficits requires attempting to disentangle “true” impairment from pre-existing proficiency differences. Arguably, this challenge forms a cornerstone of bilingual aphasia assessment, where traditional monolingual frameworks fail to capture the complex interaction between pre-stroke language abilities and acquired deficits.

These neurobiological findings make it clear that accurate assessment of bilingual aphasia must disentangle structural impairment from premorbid variability.

## 3. Assessment in Bilingual Aphasia

### 3.1. Pre-Stroke Proficiency and Post-Stroke Language Performance

The relationship between pre-stroke language proficiency and post-stroke recovery in bilingual aphasia has been debated since the earliest reported cases of multilingual aphasia. One of the central questions that has permeated the field concerns how each language is affected following focal brain damage. Historically, Ribot’s law contended that earlier-acquired languages would be more resilient to brain damage [67], whereas Pitres’ law countered that the most-used language at the time of stroke, regardless of acquisition order, would be better preserved [68,69]. This debate has persisted for over a century and remains a primary focus of bilingual aphasia research. As alluded to in the previous section, the answer to this question is not as straightforward as Ribot and Pitres originally postulated. Rather, because the neuroanatomical substrates of L1 and L2 and the degree of overlap between the two are modulated by individual factors such as age of acquisition and frequency of use, focal brain damage can have variable effects on L1 and L2 processing and language control across individuals. This variability not only complicates predictions about language outcomes post-stroke but also poses fundamental challenges for assessment and diagnosis.

The core difficulty lies in the fact that post-stroke language assessments inevitably conflate pre-stroke abilities with acquired deficits, making it difficult to isolate the effects of brain injury. Because objective data on premorbid language abilities are rarely available, clinicians and researchers must often rely on retrospective self-reports of bilingual language history and subjective ratings of pre-stroke proficiency. While these approaches are necessary, they are not without limitations. For instance, self-report measures are prone to bias and often lack the reliability and validity necessary to accurately capture language proficiency [70,71,72,73]. Consequently, the field has come to depend on a wide array of indirect and often non-equivalent metrics, each attempting to capture different dimensions of bilingual language experience. In the absence of a standardized or objective method for measuring pre-stroke proficiency, interpreting post-stroke language assessments becomes not only complex but also time-consuming and resource-intensive. As Peñaloza et al. [74] emphasized, these limitations have led many studies to adopt incomplete or inaccurate profiles of bilingual language history, potentially contributing to mischaracterizations of pre-stroke language dominance or misinterpretations of cross-language differences in post-stroke performance.

Despite these methodological challenges, convergent evidence has begun to resolve key questions about the role of pre-stroke factors in post-stroke impairment and recovery patterns. For instance, the age of acquisition (AoA) has been established as a predictor of post-stroke language performance when employing a critical threshold at 7 years [18]. Further meta-analytic evidence shows that languages learned before this age demonstrate parallel recovery patterns, while those acquired later show systematic L1 advantages [17]. Other factors, including pre-stroke language use and self-rated pre-stroke proficiency, have also been shown to modulate post-stroke performance, particularly for individuals who reported greater use and proficiency in their later acquired L2 [18]. Positive associations between pre-stroke proficiency and post-stroke language outcomes have also been observed in a range of language domains, including verbal comprehension, semantic processing, naming, and word generation in BWA, further supporting a close relationship between pre-stroke language abilities and post-stroke outcomes [75]. Moreover, evidence has revealed that pre-stroke proficiency, captured via principal components of L1 and L2 LUQ metrics, significantly predicted post-stroke lexical–semantic performance, with L1 and L2 pre-stroke proficiency explaining 33% and 38% of the variance in L1 and L2 performance, respectively [74].

Gray and Kiran [7] provided crucial early evidence by examining the association between pre-stroke proficiency and post-stroke language deficits in 19 Spanish–English BWA, finding that self-reported ability ratings in each language predicted language impairment patterns. Similarly, Muñoz et al. [76] described post-stroke profiles of four Spanish–English BWA in relation to their pre-stroke language history, identifying three distinct patterns: performance consistent with premorbid skill, performance inconsistent with premorbid skill, and variable performance inconsistent with premorbid skill. More recent work by Tschirren et al. [77] examined the effects of late L2 age of acquisition on syntactic impairment in twelve BWA, finding that although L1 and L2 aphasia severity suggested similar impairment across languages, four BWA presented with larger syntactic impairment in L2 relative to L1.

Collectively, these findings establish that pre-stroke proficiency, particularly AoA, represents a fundamental determinant of post-stroke language outcomes in BWA. However, while AoA is frequently reported as one of the strongest predictors of post-stroke impairment and recovery, this pattern may partially reflect sampling artifacts. For instance, individuals acquiring a second language before or after the age of seven often differ systematically in sociocultural background, such as childhood immersion versus adult immigration contexts [16,78]. Moreover, AoA is often easier to report retrospectively because it is typically anchored to salient life events (e.g., starting school, immigration), whereas more nuanced dimensions of bilingualism—such as exposure, use, and proficiency—require subjective judgments and are more dynamic across the lifespan [33,79]. Relying on AoA as the sole proxy for pre-stroke proficiency, therefore, risks oversimplifying the complexity of bilingual language experiences [32]. While theoretical advances increasingly emphasize the need for multifactorial approaches to estimating pre-stroke proficiency, applying these insights in clinical practice remains a significant challenge.

### 3.2. Estimating Language Proficiency in Bilingual PWA

The challenges related to quantifying proficiency hold considerable consequences for assessment in bilingual aphasia, as performance on language assessments reflects not only post-stroke impairment but also pre-stroke proficiency. Thus, interpreting assessment results requires a deep understanding of an individual’s premorbid language history [18,19,80,81,82].

However, the task of disentangling impairments related to aphasia from incomplete acquisition or language attrition prior to aphasia onset is particularly complex [12,76,81,83,84,85,86,87]. Unlike monolinguals, whose scores can be interpreted against standardized norms, bilinguals lack equivalent benchmarks due to the substantial variability in language experiences [88]. As a result, accurate diagnosis of aphasia in bilinguals necessitates individualized profiling of premorbid language proficiency in both L1 and L2.

However, estimating pre-stroke proficiency presents challenges of its own. Bilingualism is a dynamic, multidimensional construct shaped by experiential factors such as AoA, use and exposure patterns, educational history, and the larger sociocultural environments in which a language is acquired and used [15,34,78,89,90,91,92,93]. These dimensions are not static but evolve across the lifespan as environmental and lifestyle factors change [33]. Moreover, language use, dominance, and preferences may further shift post-stroke [19]. Therefore, any assessment protocols for bilingual aphasia must comprehensively characterize both pre- and post-stroke language dynamics.

Despite bilingualism being widely accepted as a continuum rather than a binary trait [78], there remains no gold-standard approach for quantifying it in a clinically meaningful or comparable way [34]. Without consensus on how to weigh or combine relevant variables, interpretation remains highly context-dependent and time-consuming. Recent methodological advances have begun to address these challenges through more sophisticated approaches to proficiency estimation. For example, rather than relying on separate measures of bilingualism, some researchers have adopted dimensionality reduction techniques to represent pre-stroke proficiency, enabling them to incorporate multiple factors such as language use, educational history, lifetime exposure, and language ability ratings [82]. This multifactorial approach has revealed that pre-stroke proficiency emerges as a complex construct, with L1 and L2 abilities mapping onto both shared and distinct bilingualism dimensions in each respective language.

#### Limitations of Current Assessment Tools

As it stands, bilingual language history questionnaires are the most commonly used tools for estimating premorbid proficiency. These instruments collect retrospective self-reports on variables such as age of acquisition, language use, educational history, and self-rated language abilities [31,34,78,80,82,94,95,96,97,98,99]. While informative, they present several measurement challenges, including reliance on retrospective self-reports, recall bias, and lack of standardized scoring procedures [35,100]. Furthermore, instruments often employ different question structures or scales to elicit responses, introducing considerable variability in how similar dimensions of bilingualism are captured.

A recent study by Dass et al. [31] underscored these challenges. The authors compared seven commonly used bilingual history questionnaires and found minimal overlap in content between instruments, as well as inconsistent classifications of bilingualism for the same individuals. In one striking example, a single participant was categorized among the “most” bilingual by two instruments and among the “least” bilingual by two others. The authors note that these discrepancies were largely driven by factors such as questionnaire length, item structure, and specific focus, such as general bilingual history versus language switching behaviors.

These findings have important implications for bilingual aphasia, where clinical decisions hinge upon accurately characterizing pre-stroke language profiles. Critically, distinct dimensions of bilingualism may be differentially relevant for interpreting post-stroke language performance. For example, not all post-stroke language switching or mixing behaviors are pathological in nature; rather, such behaviors may reflect premorbid usage patterns or could serve as compensatory strategies to navigate communication breakdowns. Without a comprehensive understanding of an individual’s pre-stroke language behaviors, experiences, and linguistic knowledge, clinicians risk misdiagnosing language impairments—potentially undermining the selection of appropriate treatment targets and recovery goals [101].

Together, these observations point to two key considerations when collecting language histories from BWA: (i) the chosen questionnaire(s) should align with the specific clinical or research questions at hand, and (ii) when feasible, using multiple tools can offer a more comprehensive and reliable profile of premorbid language proficiency [31,35,100].

A range of instruments has been developed to quantify bilingual language history, each differing in scope, structure, and the specific dimensions they measure [31]. Some commonly used tools include the Language Use Questionnaire (LUQ; [80,82], Language Experience and Proficiency Questionnaire (LEAP-Q; [97]), Bilingual Language Profile (BLP; [95,96]), Language and Social Background Questionnaire (LSBQ; [78,94]), Language History Questionnaire (LHQ3; [102]), Bilingual Switching Questionnaire (BSWQ; [99]), and Bilingual Code-Switching Profile (BCSP; [98]). These instruments differ in the extent to which they quantify language proficiency, capture exposure and use across language contexts, assess code-switching behavior, or account for sociocultural and attitudinal factors. For example, the LEAP-Q provides fine-grained self-ratings across multiple domains, while the LSBQ and BCSP focus more on switching patterns and social language use. The LUQ was specifically designed to capture both pre- and post-stroke language dynamics, enabling examination of how language patterns may have shifted following brain injury. Importantly, no single gold-standard instrument exists, and the lack of standardization complicates comparisons across studies. Therefore, researchers and clinicians must carefully select or combine tools based on the specific goals of their work to construct a more comprehensive and interpretable profile of bilingual experience—particularly in clinical populations such as bilingual aphasia, where accurate premorbid characterization is essential for diagnosis and treatment planning.

Not only is capturing pre-stroke proficiency an inherent challenge, but assessing language impairments presents unique barriers of its own. Most notably, traditional diagnostic and rehabilitation frameworks for aphasia have been overwhelmingly developed with monolingual English speakers in mind, limiting their applicability and efficacy for bilingual populations. As a consequence, standardized assessments and diagnostic frameworks often neglect key factors related to bilingualism, such as language dominance, age of acquisition, proficiency, cross-linguistic interactions, and cultural-linguistic differences. As a result, they may over- or under-estimate the extent of language impairments among BWA, mistake typical cross-linguistic influences or transfer effects as impairments arising from stroke, or overlook typological differences between a bilingual’s two languages that are critical for understanding the nature of impairments in each language [32,83,86,101,103].

Some widely used standardized language assessments—such as the Western Aphasia Battery (WAB) and Boston Naming Test (BNT)—have been made available in other languages. However, these versions are often the result of direct translations from English, which fail to account for critical cultural and psycholinguistic differences between language pairs. Consequently, such assessments may be inadequate for capturing language impairments in bilingual individuals, leading to potential misdiagnoses or underestimations of deficits [101,103]. In contrast, the Bilingual Aphasia Test (BAT; [104]) was specifically designed to be culturally and linguistically appropriate across multiple language pairs and evaluates a broad range of language domains and modalities, offering a more context-sensitive tool for assessment of bilingual aphasia. However, its clinical utility is constrained by a limited number of items per subtest and the absence of composite domain scores or an overall aphasia severity score, making it difficult to quantify impairment or track progress over time.

In contrast, the Comprehensive Aphasia Test (CAT)—a multidimensional battery assessing language, cognition, and quality of life—provides both detailed subtest scores and an overall aphasia severity score. It has been the focus of large-scale, cross-linguistic adaptation efforts [103]. These adaptations retain the CAT’s structure and scoring while developing language-specific materials that preserve key psycholinguistic properties (e.g., frequency, imageability) and ensure cultural relevance. Each version must then be standardized, normed, and validated—a resource-intensive process still ongoing in several languages. While the CAT’s broader scope and relative brevity may offer practical advantages over the BAT in some contexts, its effectiveness for bilingual assessment depends on the availability of fully validated versions in each of the speakers’ languages.

Given these limitations, clinicians and researchers working with bilingual populations must carefully consider the appropriateness of standardized tools and supplement them with alternative assessment strategies when needed. For example, picture description and other discourse tasks can elicit spontaneous language samples, allowing for qualitative and quantitative analysis of language production in each language [101]. However, these tasks must also be adapted when necessary to ensure cultural and linguistic relevance, particularly when selecting stimuli. Verbal fluency tasks provide another useful alternative, as they are quick to administer, are not constrained by visual stimuli, and offer insight into lexical access and retrieval in both languages. Moreover, simple modifications to traditional paradigms can be used to probe language control mechanisms and cross-language influences, making them especially valuable in bilingual aphasia assessment [64,105].

Building on these innovations, recent work has advocated for the integration of translanguaging frameworks to more dynamically assess language abilities in BWA [37]. Rather than treating languages as strictly separable systems, translanguaging approaches evaluate communication across the speaker’s full linguistic repertoire. In a case study of a trilingual woman with aphasia, Goral et al. [37] demonstrated that scoring performance across all languages—rather than restricting responses to the target language—yielded more accurate and ecologically valid reflections of the participant’s communicative capacity, particularly in her more impaired languages. This framework acknowledges that multilingual speakers often mix languages pragmatically and strategically. Recognizing this behavior in assessment can uncover preserved language abilities that would otherwise be overlooked. As such, translanguaging offers a promising avenue for capturing real-world communication, reducing underestimation of language abilities, and informing functionally relevant treatment planning.

In sum, accurate assessment of bilingual aphasia cannot rely solely on standardized tools developed for monolingual populations. A comprehensive, individualized approach is needed—one that reflects the dynamic nature of bilingual language use, changes across the lifespan, and sociocultural context. While instruments such as the BAT and adapted monolingual assessments provide useful starting points, they vary in scope and often miss key aspects of bilingual experience. Similarly, bilingual language history questionnaires show inconsistent results across individuals, due in part to a lack of standardization. These limitations underscore the need for flexible, multimodal assessment strategies that combine formal tests with informal, ecologically valid tasks [101]. By accounting for language proficiency, control, and cross-linguistic interaction, clinicians and researchers can better capture premorbid language profiles and support more accurate diagnoses. Ultimately, such approaches are essential for guiding personalized and culturally responsive treatment planning. The following section will explore how these diagnostic considerations shape intervention strategies and the design of effective therapy for BWA.

## 4. Treatment Approaches and Considerations

### 4.1. Mechanisms of Cross-Language Generalization

**Figure 2:**
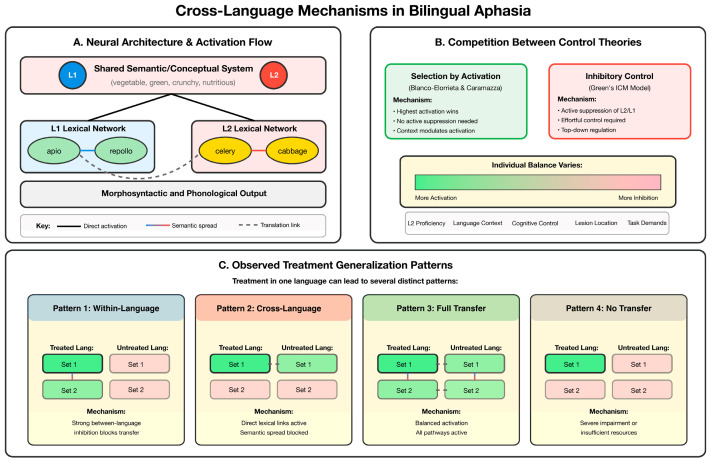
Cross-language mechanisms in bilingual aphasia. (**A**) depicts the bilingual language system with shared semantic–conceptual representations, language-specific nodes (L1, L2), and parallel lexical networks connected by translation links (dashed lines). Direct activation flows from semantic to lexical levels, with within-language semantic connections shown between related items. (**B**) presents two theoretical frameworks for language selection: selection-by-activation, where the highest activated item is selected without suppression, versus inhibitory control, requiring active suppression of the non-target language. The gradient bar imagines that individuals operate along a continuum between these mechanisms. The gray box identifies several factors that determine where an individual may fall on this activation–inhibition spectrum. (**C**) displays four distinct response patterns following treatment in bilingual aphasia. Green indicates improvement; pink indicates no improvement. Patterns demonstrate how the balance between facilitation and inhibition mechanisms, modulated by the factors in Panel (**B**), determines treatment outcomes in bilingual aphasia.

Cross-language generalization (CLG), defined as the transfer of treatment gains from a treated language to an untreated language in BWA, has emerged as a critical clinical outcome with significant theoretical and practical implications. Figure 2 summarizes the conceptual and neurocognitive mechanisms proposed to underlie CLG (Panels A–B) and illustrates four prototypical treatment-response patterns observed in bilingual aphasia (Panel C). Together, these elements provide a framework for interpreting the heterogeneous findings described in the literature and for understanding how individual differences in language experience and cognitive control shape CLG outcomes.

Early investigations into CLG produced mixed results across individual case studies and small group investigations, with some studies demonstrating CLG in cognitive and cognate-based treatments [106], while others found limited transfer in French–English bilingual aphasia [107]. Studies by Edmonds and Kiran [25] provided evidence that treatment of a premorbidly less dominant language could facilitate CLG in unbalanced bilinguals, whereas research by Kiran and Roberts [108] using semantic feature analysis treatment showed variable patterns of cross-language effects. Additional investigations continued to yield heterogeneous findings, prompting the need for synthesis to clarify patterns and predictors of CLG [32,109,110,111,112,113]. Recent meta-analytic evidence has definitively established that CLG occurs systematically, though with important constraints and moderating factors that shape its clinical potential.

Early systematic reviews attempted to synthesize these disparate findings and identify patterns in CLG outcomes. The foundational review by Faroqi-Shah et al. [114] examined 14 studies with 45 participants and documented mixed results for cross-language benefits, with approximately half of the studies showing generalization from L2 to L1 for expressive skills, while transfer from L1 to L2 comprehension showed more consistent positive outcomes. Briefly, the analyses concluded that neither age of L2 acquisition nor language typology adequately accounted for the variability in results. Building on this work, Ansaldo and Ghazi Saidi [16] reviewed 15 articles focused specifically on cross-language generalization and confirmed the potential benefits of treatment in one language on untreated languages. Critically, they demonstrated that studies comparing cognates versus non-cognates showed greater cross-language generalization for cognates, highlighting the role of shared lexical representations. However, both reviews concluded that evidence remained insufficient to determine the role of pre- and post-stroke language abilities in facilitating CLG, and neither found significant effects of overall language distance on transfer outcomes.

The largest multilingual analysis to date, conducted by Goral et al. [17] across 40 studies with 85 participants, documented significant cross-language treatment effects with small-to-medium effect sizes (g = 0.14). While these effects are statistically reliable, they remain substantially smaller than within-language treatment effects (g = 0.36), establishing a clear hierarchy in treatment efficacy patterns. Similarly, Lee and Faroqi-Shah [26] conducted a focused meta-analysis specifically examining anomia treatment in 39 BWA across 17 studies. Their analysis revealed that cross-language generalization of trained words (CLG-Tx) achieved medium effect sizes (M = 1.56), whereas CLG of semantically-related and -unrelated untrained words showed nonsignificant effects. Crucially, their regression analyses revealed that higher pre-treatment naming scores in the treated language predicted larger CLG to semantically-related words (CLG-related), whereas fewer treatment sessions predicted larger CLG to unrelated words (CLG-unrelated), suggesting that baseline lexical abilities and treatment intensity operate through distinct mechanisms to influence different types of CLG.

Interestingly, the age of acquisition emerges as the primary predictor of cross-language transfer in multilingual populations. Goral et al. [17] found that the largest CLG outcomes occurred when treatment was administered in languages acquired in adulthood (g = 0.43), followed by first-language treatment (g = 0.32), early childhood acquisition (g = 0.25), and finally later childhood acquisition (g = 0.18). The authors interpret this seemingly paradoxical finding—that later-learned languages show the strongest treatment effects—through the lens of language immersion and use patterns. In their analysis, all 11 participants treated in languages acquired during adulthood had undergone extensive immersion experiences where the later-learned language became their primary language of use prior to stroke onset. This suggests that language use and environmental exposure may modulate the effect of age of acquisition, supporting predictions by early theorists like Pitres [68] mentioned in Section 3.1 that the language most used at the time of stroke, rather than the first-acquired language, would be more likely to benefit from treatment. From our perspective, this finding underscores the dynamic nature of bilingual language organization, where patterns of language use and immersion can “override” traditional age of acquisition hierarchies in determining treatment responsiveness and cross-language transfer potential.

Next, Marte et al. [22] examined the largest single-cohort study of 48 Spanish–English bilingual individuals with post-stroke aphasia, representing the most homogeneous and largest bilingual treatment cohort studied to date. Their machine learning models achieved prediction performance for CLG with F1 scores of 0.790 ± 0.172, incorporating 16 curated features spanning demographics, language abilities, cognition, and bilingual experience. Using interpretability analyses, they identified aphasia severity in the untreated language (WAB-R AQ scores) and cognitive performance (including Raven’s Progressive Matrices) as the strongest predictors of CLG. The authors interpreted this finding as evidence that residual language function in the untreated language provides the necessary substrate for cross-language transfer, while cognitive abilities—particularly executive control mechanisms crucial for bilingual language processing—modulate the efficiency of this transfer. Their interaction analysis revealed that cognitive abilities enhance the beneficial effects of preserved, untreated language function, suggesting that domain-general control networks may facilitate transfer between language systems in contexts requiring coordination of multiple linguistic representations.

Notably, treatment-specific factors may systematically influence transfer outcomes across studies. Both meta-analytic evidence and individual investigations demonstrate that translation equivalents of trained items show significantly larger cross-language effects. Goral et al. [17] found that trained items yielded cross-language effects of g = 0.57 compared to untrained items at g = 0.35, supporting item-specific transfer mechanisms. Lee and Faroqi-Shah [26] further specified this pattern, showing that while CLG for trained words reaches medium effect sizes in bilingual populations, generalization to untrained semantically-related and -unrelated words remains highly limited. However, CLG occurs across different levels of language organization, with Goral et al. [17] finding no significant differences between within-level generalization (g = 0.29), where treatment and testing targeted the same linguistic level (e.g., both at word or sentence level), and across-level generalization (g = 0.29), where treatment targeted one level but testing measured a different level (e.g., word-level treatment with sentence-level testing). This finding suggests that cross-language transfer mechanisms operate beyond structural similarities between treatment targets and outcome measures.

Next, contrary to previous theoretical predictions, several variables showed limited impact on CLG across multilingual populations, though with important nuances. Language proficiency relationships, language distance (coded on a 10-step similarity scale), and aphasia severity failed to predict cross-language outcomes in the Goral et al. [17] meta-analysis, challenging assumptions about the role of linguistic similarity and relative language strength in facilitating transfer. The former finding was consistent with earlier reviews by Ansaldo and Ghazi Saidi [16] and Faroqi-Shah et al. [26] that found no significant effects of overall language distance. However, the narrower bilingual analyses by Lee and Faroqi-Shah [26] and Marte et al. [22] suggest that these relationships may be more nuanced, with both studies finding an influence of baseline naming or aphasia severity. Furthermore, while overall language distance may not have predicted CLG in Goral et al. [17], Ansaldo and Ghazi Saidi [16] demonstrated that cognate status—which may be conceived of as a more specific form of lexical similarity—does facilitate cross-language transfer, and Kohnert [106] found similar benefits for cognate-based treatments.

The convergent evidence from systematic reviews, meta-analytic, and machine learning approaches suggests that CLG, while modest in magnitude compared to within-language effects, represents a reliable phenomenon that can meaningfully impact bilingual treatment outcomes. Early reviews by Kohnert [115] and subsequent systematic analyses have paved the way for an increasingly sophisticated understanding of transfer mechanisms. For patients requiring functional communication in multiple languages, understanding the factors that predict and enhance cross-language transfer becomes essential for maximizing treatment efficiency and addressing real-world communication needs across linguistic contexts. The emerging evidence from Marte et al. [22] indicates that consideration of both treated and untreated language severity and cognitive assessment performance can meaningfully impact clinical decision-making for BWA, while individual case studies continue to illustrate the complexity of factors influencing transfer outcomes in specific language pairs and treatment contexts.

From a neurobiological perspective, variability in CLG outcomes can be explained by two major factors: (i) the degree of overlap in neural representations across languages and (ii) the integrity of domain-general cognitive control networks that support bilingual language processing [56]. Converging evidence from neuroimaging and lesion-symptom mapping studies supports a hybrid bilingual architecture, in which bilinguals rely on largely shared neural substrates, though the extent of overlap varies according to factors such as language typology, relative proficiency, and patterns of exposure and use [59,116,117]. Consequently, the likelihood that treatment in one language will generalize to the untreated language depends on how similarly linguistic representations are encoded within shared neural networks [56].

This overlap is strongest for conceptual–semantic representations, which are largely shared across both languages [14,59,60,116,118], though cross-language effects can also occur at other representational levels, including syntax and phonology [56]. Mechanistically, CLG is thought to arise from spreading activation, as similarly encoded linguistic forms become co-activated in parallel [59,116,117,119,120], so strengthening connections in one language can extend to the untreated language when they share similar neural encodings.

Beyond representational overlap, bilinguals rely on domain-general control networks to manage competition between lexical representations across languages. These networks comprise regions such as the dorsolateral prefrontal cortex (DLPFC), anterior cingulate cortex (ACC), and basal ganglia, which mediate processes such as selection, inhibition, and set shifting [6,8,60,61,121,122]. Intact control systems can support CLG by biasing activation toward relevant representations and help to maintain the balance between activation and inhibition of competing linguistic representations. Conversely, damage to these networks may reduce or even reverse cross-language benefits, due to increased cross-language interference [16].

Importantly, the degree of representational overlap and reliance on control mechanisms is not fixed but shaped by individual bilingual experiences, including age of acquisition, proficiency, and patterns of use and exposure. This aligns with current behavioral evidence demonstrating that bilingual language history variables are key determinants of CLG outcomes [17]. Taken together, these findings highlight that successful CLG in bilingual aphasia likely depends on the interaction between treatment factors (therapy type and treatment items), language-specific neural organization, cognitive control capacity, and individual language experience.

In sum, this evolving evidence base establishes CLG as a systematic but constrained outcome in bilingual aphasia treatment. Key predictors include age of acquisition, language use, baseline abilities in both treated and untreated languages, and cognitive control—particularly executive function [22,26]. Item-specific factors such as trained words and cognates show the strongest effects [17,26], though transfer can extend across linguistic levels. These patterns reflect a hybrid bilingual language architecture, where shared representations and domain-general control networks support activation across languages. Although effect sizes are moderate (g = 0.14–0.43), they are reliable and clinically meaningful. CLG thus offers a promising pathway for enhancing treatment efficiency in multilingual populations and deepening theoretical insight into bilingual language organization.

### 4.2. Evidence-Based Treatment Approaches

#### 4.2.1. Semantic Feature Analysis

Semantic feature analysis (SFA) represents one of the most extensively studied and theoretically grounded approaches for treating word retrieval deficits in bilingual aphasia. The treatment’s theoretical foundation rests on principles of spreading activation within semantic networks [123]. It leverages the interconnected nature of conceptual representations to strengthen damaged pathways or create new connections between semantic and lexical systems. In the standard SFA protocol, a clinician presents a picture of a target word. The patient then systematically generates or reviews semantic features related to that word. These features may include its category, use or function, physical properties, location, and personal associations, among others [124]. This structured activation of semantic features is thought to strengthen connections within the semantic network through repeated practice, improving access to the target word and potentially to semantically-related concepts.

Notably, in bilingual individuals, this spreading activation mechanism operates within theoretical frameworks that posit shared conceptual representations across languages. Contemporary models of bilingual lexical organization (e.g., [125,126,127]) and computational implementations (e.g., Multilink [128]; see [129] for a review) suggest that while lexical forms are language-specific, conceptual representations are largely shared between languages. This architectural arrangement provides the theoretical basis for cross-language generalization in SFA: strengthening semantic–lexical connections in one language can potentially facilitate access to translation equivalents and semantically-related words in the untreated language through their shared conceptual substrate.

Gray and Kiran [7] provided theoretical insights into the relationship between semantic and lexical deficits in bilingual aphasia that help explain SFA’s differential effects. Their analysis of 19 Spanish–English BWA revealed two distinct impairment patterns: patients with primarily lexical-level deficits who lost similar amounts of language ability in both languages and those with combined semantic–lexical deficits showing differential impairment across languages. This theoretical framework suggests that SFA may be particularly effective for individuals with preserved semantic systems but impaired lexical access, as the treatment can leverage intact conceptual representations to strengthen degraded lexical connections.

Furthermore, the Rehabilitation Treatment Specification System (RTSS) framework provides a structured lens for understanding SFA’s mechanisms of action in bilingual contexts [27] and thus treatment outcomes. Within this framework, the primary treatment target of SFA is word retrieval accuracy, with the treatment ingredients comprising the systematic generation and review of semantic features, repeated pairing of features with target labels, and the use of visual aids to support feature generation [24]. The mechanism of action posits that directly targeting the semantic system strengthens connections between trained items and related concepts, thereby improving lexical access not only to trained items but potentially to semantically-related untreated items.

The meta-analysis by Lee and Faroqi-Shah [26] provides the most rigorous synthesis to date of SFA outcomes in bilingual aphasia. Analyzing data from 17 published studies encompassing 39 BWA, they found that semantic treatments (predominantly SFA, represented in 13 of 17 studies) produced medium effect sizes for trained items in both L1 (TE = 8.36) and non-L1 (TE = 1.63). However, the analysis revealed constrained generalization patterns, with within-language generalization to semantically-related, untrained words showing small effects (WLG-related = 0.63) and minimal generalization to unrelated words (WLG-unrelated = 1.56). Cross-language generalization effects were similarly modest for both trained word translations (CLG-Tx = 0.68) and semantically-related, untrained words (CLG-related = 0.63).

These findings align with prior individual studies that have systematically examined SFA outcomes in bilingual populations. Edmonds and Kiran [25] provided early evidence of SFA’s efficacy in Spanish–English bilinguals, demonstrating improvements in trained items with variable patterns of within- and cross-language generalization. Their work established that treatment in either language could facilitate retrieval in both languages, though the magnitude and consistency of cross-language effects varied considerably across participants. Subsequent investigations by Kiran and Roberts [108] and Kiran et al. [130] with larger samples confirmed these patterns, showing robust within-language treatment effects with more variable cross-language outcomes.

Recent investigations have begun to identify the active ingredients that drive SFA’s effectiveness in bilingual contexts. Li et al. [27] conducted a detailed analysis of treatment components in 12 Mandarin–English bilinguals receiving modified SFA (mSFA) for both nouns and verbs. Their findings revealed that spontaneous naming emerged as crucial for trained item improvement, while feature analysis and sentence production predicted generalization to semantically-related items. Importantly, all treatment steps (i.e., naming practice, feature analysis, word association, and sentence production) contributed significantly to cross-language generalization, suggesting that each component plays a vital role in facilitating transfer across languages. The authors theorized that feature analysis and word association enhance connections between conceptual representations and lexical forms in both languages, while repeated naming practice reinforces pathways between the conceptual system and lexical representations across languages.

The question of optimal treatment parameters in SFA has been examined through multiple analytical approaches using data from a large randomized controlled trial of Spanish–English bilinguals [12]. Scimeca et al. [23] examined multilevel predictors—factors operating at different levels, including intervention-level (e.g., treatment language, dosage), individual-level (e.g., age, severity), and stimulus-level (e.g., word frequency, phonological complexity), of treatment response in participants from this trial who received 40 h of SFA. Their analysis revealed that individuals receiving therapy in their L1 showed greater improvement in the treated language, with higher pre-treatment naming scores predicting better treatment response, challenging earlier assumptions about treating the weaker language to maximize cross-language transfer and suggesting that residual language abilities may provide the necessary substrate for treatment gains. Following completion of the full RCT, Marte et al. [22] applied machine learning approaches to the complete dataset of 48 participants, incorporating eight feature sets: demographics (education, age, months post-onset), Language Use Questionnaire data, severity measures for both languages (WAB-R Aphasia Quotient), linguistic performance in treated and untreated languages, cognitive assessment scores, and patient-treatment language characteristics (treatment language, L1/L2 status). The top-performing models achieved strong predictive accuracy for both treated language improvements (F1 = 0.77) and cross-language generalization (F1 = 0.79). Interpretability analyses extended Scimeca et al.’s [23] findings by identifying aphasia severity in the untreated language and cognitive performance as the strongest predictors of cross-language transfer. Together, the studies suggest that both language-specific factors (baseline naming ability, untreated language severity) and domain-general factors (cognitive performance) converge to influence SFA-based treatment outcomes in bilingual aphasia.

Building on these insights, Russell-Meill et al. [28] examined the breadth of generalization patterns in this same cohort of 48 Spanish–English bilinguals. Using a framework that distinguishes transfer effects by proximity to the treatment focus, from direct transfer (same skill domain) to near transfer (related domains) to far transfer (distant domains), they documented a gradient of treatment effects following SFA. The findings revealed robust direct transfer to untrained naming items, near transfer to semantic processing tasks requiring similar underlying processes (including synonym judgment and semantic association), and far transfer limited to global language ability as measured by the Western Aphasia Battery. Crucially, improvements did not extend to nonverbal abstract reasoning, indicating that while SFA can promote broad language improvements across both languages and multiple linguistic domains, its effects remain domain-specific rather than generalizing to broader cognitive abilities. Overall, the pattern aligns with the cognitive training literature consensus [131,132,133] that treatment effects diminish as the distance between trained and assessed domains increases, with language treatments potentially showing a unique capacity for transfer within interconnected linguistic systems due to their reliance on both linguistic and domain-general cognitive processes.

The accumulated evidence establishes SFA as an effective approach for treating word retrieval deficits in bilingual aphasia, with several key principles emerging. First, treatment effects follow a predictable gradient, with the most robust improvements for directly trained items, diminishing for within-language generalization to semantically-related words, and smallest for cross-language transfer [26], extending beyond naming to show robust direct transfer, selective near transfer to semantic processing tasks, and far transfer limited to global language abilities but not domain-general cognition [28]. Second, individual variability in treatment response appears driven by a convergence of factors, with machine learning analyses revealing that cross-language generalization is best predicted by aphasia severity in the untreated language and cognitive performance [22], while within-language gains are enhanced by higher baseline naming abilities and L1 treatment [23], potentially interacting with underlying impairment profiles where individuals with preserved semantic systems but impaired lexical access may respond more favorably to SFA’s mechanism of strengthening lexical–semantic connections [7]. Third, the multi-component nature of SFA appears crucial for maximizing outcomes, as Li et al. [27] demonstrated that while spontaneous naming drives improvement in trained items, all treatment components (i.e., naming practice, feature analysis, word association, and sentence production) contribute significantly to cross-language generalization, suggesting that the full protocol is necessary for facilitating transfer across languages. These findings underscore the importance of comprehensive baseline assessment incorporating linguistic, cognitive, and bilingual experience measures [12] to identify optimal candidates and treatment parameters, ultimately maximizing the potential for meaningful improvements across both languages in bilingual aphasia rehabilitation.

#### 4.2.2. Verb Network Strengthening Treatment

While the majority of treatment research in bilingual aphasia has focused on noun retrieval, Verb Network Strengthening Treatment (VNeST) represents an important extension of semantic-based approaches to verb deficits. Developed by Edmonds et al. [134], VNeST targets verb retrieval in sentence contexts by leveraging the theoretical principle that verbs and their thematic roles (agents and patients) are co-activated during lexical retrieval. The protocol involves systematic generation and discussion of agent-patient pairs for target verbs, e.g., given “measure,” participants might generate “carpenter-wood” or “chef-ingredients,” then elaborate on why, where, and when these activities occur. This approach theoretically strengthens the verb’s semantic representation by activating multiple exemplars and contexts, facilitating more robust retrieval pathways through spreading activation. Unlike noun-focused treatments that primarily target object concepts, VNeST capitalizes on verbs’ inherent relational nature and their central role in sentence construction.

Evidence for VNeST in bilingual populations remains notably sparse, with only a handful of studies examining its efficacy across language pairs. Lerman et al. [112] examined VNeST effects in two English–Hebrew bilingual males with chronic aphasia (5–6 years post-stroke), using consecutive treatment blocks where each participant received approximately 29 h of treatment in each language. Both participants had moderate expressive aphasia in English and severe expressive aphasia in Hebrew. Their findings revealed stronger within-language generalization in the less-impaired L1 English than in Hebrew, with improvements observed primarily in sentence construction and discourse production. Cross-language generalization was limited overall, with some effects observed from English to Hebrew but minimal transfer from Hebrew to English. The authors interpreted these asymmetrical patterns as supporting both the Revised Hierarchical Model’s predictions about differential connectivity strengths and theories of lingering suppression during cross-language processing.

Next, Li et al. [113] examined VNeST in two Mandarin–English bilinguals with chronic aphasia, providing insights into how typological differences influence treatment outcomes. Their 10-week Mandarin VNeST treatment protocol, delivered via videoconference, revealed that both participants showed within-language generalization to semantically-related untrained verbs in Mandarin. However, cross-language generalization patterns differed: one participant (P1) showed a larger magnitude of improvement in English untrained items than English trained items (suggesting cross-language interference), while the other (P2) showed more typical cross-language transfer patterns. The study highlighted the structural differences between languages, noting that Mandarin verbs lack the morphological complexity found in English (no tense, person, or number markers), which may influence how treatment effects manifest across languages. Despite these cross-linguistic differences in verb morphology, both participants demonstrated similar verb–noun dissociation patterns in both languages, suggesting shared neural substrates for grammatical category processing. Error analysis revealed decreasing semantic errors in both participants across trained and untrained conditions. Notably, for P1, cross-linguistic errors (non-target language intrusions) decreased during Mandarin probes while increasing during English probes, suggesting that intensive training in Mandarin may have temporarily affected language control mechanisms. The authors interpreted this as potentially reflecting incomplete inhibition of the trained language during untrained language production, warranting further investigation into how verb network strengthening in one language influences control processes in bilingual speakers.

The cross-language generalization patterns following VNeST appear more complex and less predictable than those observed with noun-focused treatments. While SFA studies consistently show modest but reliable cross-language effects for trained items and their translations [26], VNeST outcomes vary dramatically across individuals and language pairs. This variability may reflect fundamental differences in how verbs are represented and processed across languages, i.e., verbs must encode not only semantic information but also argument structure, morphosyntactic features, and language-specific constraints that may share little overlap between languages [135,136]. For instance, the Mandarin–English bilinguals in Li et al. [113] faced the challenge of transferring gains between a language with minimal verb morphology (Mandarin) and one with complex tense, aspect, and agreement systems (English), potentially limiting the utility of strengthened semantic networks when syntactic demands differ so substantially. These findings suggest that while VNeST offers a theoretically motivated approach for targeting verb deficits through thematic role activation, its application in bilingual contexts may require consideration of cross-linguistic differences in verb representation and processing, with treatment outcomes likely influenced by the specific structural properties of each language pair.

#### 4.2.3. Phonological Components Analysis

Phonological components analysis (PCA) represents the phonologically-oriented counterpart to semantic-based treatments, developed by Leonard et al. [137] as an adaptation of SFA principles to target phonological rather than semantic networks. The standard protocol involves five consecutive phonological cues: identifying the first sound (“What sound does it start with?”), the final sound (“What sound does it end with?”), the number of syllables (“How many beats does the word have?”), providing a first sound associate (“What other word starts with the same sound?”), and generating a rhyme (“What does this rhyme with?”). This structured approach theoretically strengthens phonological representations through repeated activation and manipulation of sound-based features, facilitating retrieval through spreading activation within the phonological network.

The cognitive demands of PCA are particularly relevant for bilingual populations. Unlike monolinguals, bilinguals must manage interactions between phonological systems from separate languages. Since PCA’s protocol requires considerable metalinguistic awareness and executive control to consciously analyze and manipulate phonological structures, these demands could, in principle, enhance treatment outcomes for BWA, as bilingual experience has been linked to more flexible phonological representations and heightened control over language selection (e.g., [56]).

Evidence for PCA in bilingual aphasia, however, remains limited. A single study by Masson-Trottier et al. [138] compared French-PCA effects in four monolingual and four bilingual French–English persons with chronic aphasia, revealing a potential bilingual advantage in treatment outcomes. Following 15 h of treatment, bilingual participants demonstrated superior outcomes across multiple measures: stronger evidence of acquisition for treated items, greater improvements in untreated item naming, and significantly larger gains in narrative discourse production. The authors also performed a neuroanatomical analysis using imaging data, finding that bilingual participants showed significantly greater cortical thickness in right hemisphere regions associated with cognitive control (inferior frontal gyrus, middle frontal gyrus), which correlated with better Flanker task performance. The authors proposed that this structural neural reserve may enable bilinguals to more effectively utilize PCA’s cognitively demanding protocol.

Despite these promising findings, the efficacy of phonologically-based treatments for promoting CLG remains underspecified. According to Nadeau’s [56] population encoding framework, phonological treatments have the potential to facilitate CLG only when there is significant overlap in phonological sequences across a bilingual’s two languages. This constraint stands in contrast to semantic-based interventions, which benefit from extensively shared conceptual representations between languages and may therefore produce more consistent patterns of CLG. For bilinguals whose language pairs are phonologically distant, phonologically-based treatments such as PCA may offer limited cross-language benefit. Therefore, clinicians must carefully consider the degree of phonological similarity between languages when deciding the appropriateness of these treatment approaches for BWA. This is particularly true if cross-language gains are a primary goal of therapy, as treatment gains may be largely confined to one language when there is minimal overlap.

Finally, combining phonological- and semantic-based treatments may provide a unique opportunity to promote CLG in BWA whose language pairs contain many cognates—words that overlap in both form (phonology) and meaning (semantics) (e.g., *family* and *familia* in English and Spanish). Prior work has shown that cognates and cognate-based treatments can lead to robust CLG among BWA, likely due to the high degree of overlap across multiple levels of linguistic representation (i.e., semantic and phonological) [16,106]. Targeting these cross-level redundancies may therefore maximize both within- and cross-language recovery. However, this facilitative effect is not universal, and evidence suggests that it can be attenuated or even reversed in individuals with damage to cognitive control networks [16,139,140]. In such cases, impaired control mechanisms may amplify cross-language interference, particularly for highly overlapping lexical representations, due to disruptions in the balance between activation and inhibition of competing lexical candidates [139,140,141].

In summary, PCA offers a theoretically grounded, cognitively engaging approach to strengthen phonological representations, but its application in bilingual aphasia remains underexplored. Evidence suggests that PCA may be most effective for bilinguals with greater cognitive control resources and high phonological overlap between languages, particularly when incorporating cognate-rich lexical targets. However, as discussed in Section 2.2, bilingual language processing depends on the integrity of shared neural substrates and the efficiency of control networks (e.g., dorsolateral prefrontal cortex, anterior cingulate cortex, and basal ganglia) that manage competing representations. When these control systems are compromised, the benefits of phonological treatments—and especially cognate-based interventions—may diminish or even reverse, leading to cross-language interference.

Future research should systematically evaluate PCA outcomes across bilingual populations with varying language profiles, examining how language typology, cognitive control network integrity, and treatment design interact to shape recovery. Moreover, integrating phonological and semantic treatment strategies may be particularly promising for bilinguals whose language pairs contain abundant cognates, offering a pathway to maximize both within- and between-language generalization. Establishing evidence-based guidelines for when and how PCA should be applied in bilingual aphasia rehabilitation will require linking behavioral treatment outcomes with neurobiological markers of language representation and control, thereby connecting clinical practice with the neural mechanisms outlined in Section 2.2.

#### 4.2.4. Discourse and Innovative Approaches

The traditional constraint of single-language production during aphasia assessment and treatment increasingly appears misaligned with the communicative reality of multilingual individuals. Recent theories and investigations have begun to challenge this monolingual bias, proposing approaches that embrace the natural linguistic behaviors of bilinguals, inclusive of code-switching, language mixing, and flexible language choice. These emerging paradigms reflect a shift from viewing cross-linguistic intrusions as necessarily pathological to recognizing them as potential compensatory strategies that leverage the full linguistic repertoire available to bilingual speakers. The theoretical impetus for this reconceptualization stems from converging evidence that strict language separation imposes additional cognitive control demands on individuals already struggling with lexical retrieval, potentially masking preserved abilities and limiting functional communication outcomes.

The mechanistic rationale for these approaches draws from multiple lines of empirical evidence. Mooijman et al. [142] conducted an investigation of voluntary language switching in Dutch–English BWA, comparing three conditions: single-language naming, cued language switching, and voluntary language choice. Their results revealed that voluntary language choice yielded significantly higher accuracy (84.9%) compared to single-language Dutch (79.7%) or English (73.1%), with faster response times as well. Crucially, their item-level analyses demonstrated that participants strategically selected languages based on lexical accessibility, choosing Dutch for 68% of items but switching to English when it offered easier retrieval. This pattern aligns with Carpenter et al. [64], who examined verbal fluency in Spanish–English bilinguals across similar conditions. Their participants achieved the highest scores in the self-switch condition (allowing free language choice), followed by single-language conditions, with the poorest performance in forced-switching conditions where language choice was constrained. The cognitive mechanism, therefore, appears straightforward: when lexical items in a would-be non-target language no longer serve as competitors requiring suppression but instead become pragmatically acceptable word choices, the cognitive control demands diminish substantially, freeing resources for successful retrieval.

Language mixing patterns in bilingual aphasia provide additional insights into these compensatory mechanisms. Goral et al. [65] examined variation in language mixing across 11 multilingual individuals with aphasia during connected speech production, finding that mixing patterns systematically reflected lexical retrieval difficulties. Participants with greater aphasia severity demonstrated more frequent language mixing, while those with lower post-stroke proficiency in a given language showed increased mixing when attempting to speak that language. Critically, mixing was not random but followed predictable patterns, with participants mixing more during tasks requiring specific lexical retrieval (picture description) compared to less constrained narrative tasks, and avoiding mixing languages unknown to their interlocutors, demonstrating preserved pragmatic recognition. Lerman et al. [111] provided a fine-grained analysis of these patterns in a Hebrew–English bilingual with non-fluent aphasia, revealing that language mixing occurred more asymmetrically: significantly more Hebrew intrusions when speaking English (the participant’s weaker language) than English intrusions when speaking Hebrew. Moreover, function words showed higher mixing rates than content words, and single-word naming elicited more mixing than connected speech, linking mixing behavior to a gradient of lexical retrieval difficulty.

Finally, the translanguaging framework represents a reconceptualization of these phenomena. Goral et al. [37] operationalized this approach in their assessment of a trilingual person with aphasia across English, German, and Farsi, comparing traditional single-language scoring with translanguaging scoring that credited responses regardless of language used. Object naming accuracy in German improved from 53.33% to 76.67%, action naming from 33.33% to 48.15%, and narrative relatedness scores showed dramatic gains as well. The participant’s mixing patterns revealed sociolinguistic competence as well, with the participant using English while speaking German with German–English bilinguals, but never using German with Farsi–English bilinguals. Interestingly, mixing decreased as linguistic distance increased, with the authors attributing more English–German mixing than English–Farsi mixing due to greater structural overlap. The authors argue that translanguaging assessment reveals preserved abilities masked by artificial monolingual constraints, as lexical items from non-target languages serve not as errors but as successful communication when interlocutors share those languages.

The converging approaches of voluntary language switching, strategic code-switching, and translanguaging assessment reconceptualize bilingual aphasia assessment and rehabilitation. Rather than viewing language mixing as pathological interference requiring suppression, they recognize it as adaptive behavior that maximizes communicative success given available resources. As Lerman et al. [111] note, BWA mix languages more frequently in demanding contexts, with mixing being reasonably interpreted as a compensatory function for word-retrieval difficulties. Clinical implications include, for example, therapy that explicitly trains strategic language switching as a functional tool, to training patients on when and how to leverage their full, preserved lexicon. Hameau et al. [143] extend this further, proposing that code-switching could serve as a self-cueing strategy even in monolingual contexts, where switching momentarily to another language might facilitate retrieval in the target language through spreading activation across interconnected lexical networks. Moving away from enforcing language separation to encouraging flexible translanguaging aligns treatment with the lived communicative experiences of bi- and multilingual individuals and potentially unlocks preserved abilities that traditional monolingual approaches inadvertently suppress.

While current evidence-based treatments demonstrate measurable benefits for bilingual populations, several limitations constrain their clinical impact, such as the fact that individual variability in outcomes is poorly understood, and access to bilingual-competent clinicians remains limited. Emerging technological innovations offer potential solutions to these challenges through more precise prediction of treatment outcomes, automated assessment capabilities, and scalable delivery platforms.

## 5. Future Directions in Bilingual Aphasia Rehabilitation

Advances in bilingual aphasia research now intersect with breakthroughs in artificial intelligence, neurotechnology, and assessment methodology, creating unprecedented opportunities for personalized and ecologically valid rehabilitation. This section examines emerging directions that leverage these convergent developments: (1) computational approaches for predicting bilingual recovery patterns; (2) automated assessment and adaptive therapy platforms; (3) portable neurotechnologies for real-time monitoring of neural activity; and (4) naturalistic paradigms that capture authentic bilingual communication. Together, these innovations collectively represent a shift toward more effective, accessible, and culturally responsive clinical care for diverse bilingual populations.

### 5.1. Machine Learning and Computational Approaches

#### 5.1.1. Predictive Modeling in Bilingual Aphasia

Machine learning studies in monolingual aphasia reveal converging evidence for a hierarchy of predictive value in recovery and outcome modeling. Demographic and behavioral features establish a baseline of predictive accuracy; lesion topology derived from structural MRI further improves performance [144,145]; network-level connectivity from diffusion MRI or tractography adds additional gains [146]; and, in some cases, functional measures from resting-state fMRI can outperform behavioral predictors [147]. The strongest results to date come from multimodal neuroimaging approaches integrating structural MRI and resting-state fMRI with behavioral data [20]. However, these advances are based almost entirely on monolingual English-speaking cohorts, and models trained in such populations do not necessarily generalize to bilingual patients. For example, Hope et al. [38] demonstrated that prognostic models trained exclusively on monolingual data systematically overestimated bilingual performance on most language tasks, despite shared lesion–deficit associations, with bilinguals showing greater sensitivity to damage in the same regions. These findings underscore the need for predictive frameworks designed from the outset to capture cross-language interactions, premorbid proficiency, and bilingual-specific patterns of brain–language reorganization. Early work toward this goal has emerged from two complementary approaches—mechanistic simulation and data-driven statistical learning—each offering distinct insights.

One of the first mechanistic frameworks developed for bilingual aphasia prediction was BiLex, a computational model originally designed to simulate premorbid and post-stroke bilingual lexical access as a function of AoA and lifetime language exposure [19]. A later extension incorporated lesion modeling and simulated retraining, enabling retrospective prediction of therapy-induced naming gains in both treated and untreated languages for 13 Spanish–English patients with bilingual aphasia [42]. Leave-one-out cross-validation showed that by the fourth treatment session, BiLex predicted the magnitude of treated-language gains, with R^2^ values ranging from 0.54 to 0.82. Although cross-language gains were generally underestimated, the model still captured key generalization patterns, achieving 100% specificity (7/7 true negatives) and approximately 80% sensitivity (4/5 true positives) in detecting whether any CLG would occur. Building on these retrospective findings, a recently concluded randomized controlled trial prospectively evaluated BiLex-generated treatment language recommendations in 48 Spanish–English bilingual aphasia patients ([12]; results forthcoming).

The same trial data were also used to support a complementary, fully data-driven approach. Marte et al. [22]—discussed further in Section 4.1 and Section 4.2—applied supervised learning to identify predictors of recovery directly from diverse clinical variables. Their findings underscore how models shaped by patterns in clinical data can capture individual variability in recovery. Emerging from this work are several foundational principles that may support the design of more effective bilingual predictive models:

First, models must account for outcomes across both languages. While monolingual frameworks predict recovery within a single language, bilingual models must capture the complex dynamics of recovery across two languages. Marte et al. [22] addressed this by developing separate classifiers for treated language improvement (TLI) and CLG, identifying distinct predictors for each: TLI was driven by treated-language severity, education, and cognition, while CLG was more closely linked to untreated-language severity and executive function.

Second, bilingual models must incorporate individualized language history and cognitive factors. Bilingual assessment requires contextualizing performance against pre-stroke proficiency, which varies widely across individuals. Marte and colleagues [22] addressed this by integrating factors such as age of L2 acquisition, usage patterns, and self-rated proficiency to anchor predictions to personal baselines. Domain-general cognition also emerged as a core predictor, particularly for cross-language transfer outcomes, underscoring its centrality in bilingual recovery.

Finally, interpretability is especially critical in bilingual models. Because bilingual outcome prediction requires high-dimensional feature spaces—encompassing premorbid language history, proficiency, cognitive function, and usage patterns—it is imperative to understand which features drive predictions. Without this transparency, outputs cannot be meaningfully interpreted to advance theoretical knowledge or applied to individualized care. Marte et al. [22] addressed this by using SHapley Additive exPlanations (SHAP) analyses, which identify how much each feature contributes to a given prediction. This approach made it possible to verify that the model’s decisions were aligned with clinically relevant factors, thereby supporting both theoretical and clinical utility.

Despite these promising initial results, substantial opportunities remain for advancing predictive modeling in bilingual aphasia. Current bilingual models have relied primarily on behavioral and demographic features, whereas monolingual studies increasingly incorporate structural and/or functional neuroimaging data to improve performance (e.g., [20,144,145,147]). Additionally, Marte and colleagues [22] focused exclusively on treatment response prediction, leaving spontaneous recovery patterns unexplored despite evidence that cross-language interactions may fundamentally alter natural recovery trajectories [148,149,150,151]. Further, like their monolingual counterparts, these existing bilingual models also lack external validation and measures of confidence in model predictions, critical methodological gaps that must be addressed before clinical deployment.

Future investigations would benefit from pursuing several parallel developments. These could include incorporating neuroimaging predictors while examining diverse language pairs beyond Spanish–English populations (e.g., [152,153]; also see [45,93]), developing standardized assessment protocols for bi- and multilingual neuroimaging studies [154,155], and advancing both mechanistic simulation frameworks and statistical machine learning approaches using the bilingual-specific modeling principles outlined here. Finally, as these predictive modeling efforts continue, they must be complemented by innovations in outcome measurement to ensure that prognoses reflect clinically meaningful targets. A growing area of research is the development of automated diagnostic tools capable of capturing the dynamic realities of bilingual language use in more ecologically valid contexts.

#### 5.1.2. Automated Assessment Technologies

In addition to their role in outcome prediction, machine learning-based methods are increasingly being applied to diagnostic assessment. These automated technologies offer promising avenues for scalable and language-sensitive evaluation, with the potential to address the diagnostic challenges for bilingual individuals outlined in Section 3. However, significant hurdles remain in adapting these systems for bilingual users, particularly given the complexities of code-switching, unbalanced language dominance, and culturally appropriate assessment frameworks.

Although adaptation to bilingual contexts remains complex, recent progress in speech technology and natural language processing has significantly advanced monolingual aphasia assessment. A growing body of work has demonstrated the potential of automatic speech recognition (ASR)-based pipelines to support aphasia detection and characterization. Wagner et al. [43] used ASR-derived linguistic features to detect aphasia (F1 = 0.99) and classify subtypes (weighted F1 = 0.91; range = 0.78–0.98), achieving performance comparable to clinician agreement. Tang et al. [156] extended this line of work by training a joint model that simultaneously transcribes and classifies speech, improving diagnostic accuracy (97.3%) and reducing transcription error rates by 11%. Sanguedolce et al. [157] further improved transcription quality by fine-tuning a large-scale ASR model on post-stroke speech, showing robust generalization to unseen speakers and corpora. Building on these foundations, Perez et al. [158,159] developed models that detect phonemic (F1 = 0.64–0.72), neologistic (F1 = 0.61–0.69), and semantic (F1 = 0.25–0.38) paraphasias directly from audio, enabling fine-grained speech error profiling without manual transcripts. Together, these advances represent a shift toward detailed, accurate, and clinically meaningful ASR tools for aphasia assessment.

In addition to ASR tools, large language models (LLMs) have broadened the scope of automated assessment by enabling analysis of discourse-level features that align with clinical markers of agrammatism and fluency deficits, such as surprisal (a measure of how unexpected a word is given the preceding context; [160]). Rezaii et al. [161] demonstrated the clinical value of this approach by using GPT-based embeddings to cluster 78 individuals with primary progressive aphasia, identifying the three canonical PPA variants (88.5% diagnostic agreement) and achieving high accuracy in supervised subtype classification (97.9%). These findings suggest that LLM-derived features can reveal subtle language patterns that may escape manual analysis, offering a scalable path toward more precise diagnostics. However, extracting these insights within a single language is only part of the challenge, and growing attention has turned to whether such tools can generalize across languages.

Automated assessment tools that use ASR pipelines and/or pretrained language models for aphasia assessment show early promise for cross-linguistic generalization, though results vary. Notably, Chatzoudis et al. [162] trained an ASR-based aphasia assessment system in English and tested it directly on other languages without additional training, attaining high accuracy in English (up to 97%) but lower performance in French (78%) and Greek (74%). While most other studies to date have relied on manual transcriptions rather than direct ASR, they nevertheless demonstrate important precedents for cross-linguistic adaptation in automated aphasia assessment. For example, Qin et al. [163] reported 83% accuracy when applying a Cantonese-trained model directly to Mandarin speech without any Mandarin training data (zero-shot transfer), compared to 98% accuracy within Cantonese [163]. Additionally, Balagopalan et al. [164] used domain adaptation: they trained a model on English and adapted it to French and Mandarin by making small language-specific adjustments using limited target-language data. This approach boosted aphasia classification accuracy in both languages (10% and 8% increase in F1 scores for French and Mandarin, respectively, over monolingual baselines), showing that even modest tuning can reduce performance loss when applying monolingual models to new languages.

Although these studies show that cross-language adaptation is possible, they do not yet tackle the more nuanced diagnostic demands of bilingual aphasia. Most current systems remain poorly suited for bilingual speakers. Trained primarily on monolingual data, they assess each language in isolation—an approach that, like traditional clinical assessment methods, risks the misinterpretation of a bilingual individual’s less proficient language as atypical or impaired [111,165]. Prognostic models have likewise been shown to misestimate bilingual outcomes [38], and standardized, clinically validated diagnostic frameworks that integrate both languages into a single normative profile are still lacking [165], leaving crucial bilingual language dynamics beyond the reach of current automated assessment tools.

These limitations stem from how languages interact within the bilingual speaker—not merely from language-to-language differences—and manifest across multiple levels [48]. Cross-language phonemic influences, such as tonal contrasts, timing mismatches, or overlapping segmental inventories, can create atypical acoustic patterns that may strain monolingual ASR systems. Even fine-tuned models exhibit substantially higher error rates on aphasic than on non-aphasic speech—roughly twofold or more in practice [157]—a gap that may widen in bilingual contexts.

These assumptions are further disrupted in bilingual connected speech, where borrowing, code-switching, and self-repair produce patterns that blend grammars and defy monolingual norms. Critically, there are no widely validated normative baselines for these bilingual speech behaviors, making it difficult to determine whether a pattern reflects impairment, typical bilingual variation, or adaptive compensation (e.g., [34,35,90]). Without such baselines, both clinical evaluators and ASR systems risk misclassifying normal bilingual variation as pathological or overlooking impairment entirely.

Addressing these limitations will require both conceptual and technical innovation. Priorities may include developing clinician-annotated bilingual corpora that capture normative variability, building L1- and L2-specific severity models, implementing language-sensitive feature extraction pipelines, and validating automated outputs (e.g., severity scores, error profiles) against clinician-rated bilingual data. While current systems report strong monolingual accuracy, they are likely not yet capable of supporting accurate diagnosis in bilingual aphasia.

Automated assessment platforms establish a foundation for personalized diagnostics and provide a natural bridge into digital therapy systems tailored to individual impairment profiles.

#### 5.1.3. Personalized and Adaptive Digital Therapy Platforms

Innovations in predictive modeling (Section 5.1.1) and automated assessment (Section 5.1.2) naturally extend to adaptive digital therapy platforms, which use real-time performance data—often derived from the same analytic pipelines used for diagnosis—to adjust treatment difficulty, target selection, and feedback without continuous clinician oversight. In monolingual contexts, adaptive systems yield measurable benefits across domains: improved overall aphasia severity [166]; item-specific gains in spoken-word comprehension [167] and in reading accuracy and speed [168]; and additional improvements in a range of language and cognitive domains with consistent practice [40,41].

However, as with predictive modeling and assessment, current adaptive therapy platforms are constrained by single-language frameworks [30,169], limiting their accuracy and flexibility for bilingual speakers. Performance metrics are typically language-specific, risking misinterpretation of correct productions in the non-target language as errors, and overlooking the importance of cross-language transfer. Without accounting for bilingual-specific variables such as code-switching, cognate facilitation, or differential language dominance, these systems cannot fully align therapy progression with the realities of bilingual recovery.

Integrating bilingual principles into adaptive platforms could address these gaps. In semantic-based interventions, for example, responses in either language (e.g., *gato* or *cat*) could be credited as evidence of conceptual access. In lexical retrieval tasks, correct productions in the non-target language could be treated as indicators of CLG or compensatory retrieval, informing adaptive feedback and progression [37,170]. Well-documented cognate facilitation effects could also be systematically incorporated to modulate task difficulty [16,115,171], while advances in predictive modeling could guide strategic treatment language selection across therapy stages [12,22,42].

However, while adaptive therapy platforms refine intervention based on behavioral performance, they only capture the observable outputs of recovery. Neurotechnologies add a complementary dimension to this work by directly measuring brain activity, revealing the mechanisms that underlie language use, impairment, and change. These tools extend assessment into real-world settings, offering a more ecologically valid understanding of bilingual aphasia.

### 5.2. Neuroscience in Real-World Contexts

#### 5.2.1. Wearable Neuroimaging for Naturalistic and Clinical Neuroscience

In recent years, neuroscience research has increasingly moved beyond the confines of highly-controlled laboratory experiments toward more ecologically-valid paradigms that capture brain function as it unfolds during complex, real-world activities. This movement toward neuroscience in real-world contexts has the potential to transform our understanding of how the brain supports dynamic behavior and cognition and creates opportunities for translational research that bridges the gap between scientific discovery and clinical application. By integrating behavioral measures with functional neurotechnologies, researchers can better characterize neural, cognitive, and behavioral changes in post-stroke aphasia, improving diagnostic precision, tracking recovery, and informing individualized treatment approaches to improve real-world communication outcomes.

In post-stroke aphasia, functional neuroimaging is a powerful tool for identifying residual brain function, predicting recovery potential, and assessing treatment responsiveness [172,173,174,175]. Functional near-infrared spectroscopy (fNIRS) and electroencephalography (EEG) are two noninvasive, cost-effective, and portable modalities that are increasingly being used to assess brain activity in clinical and naturalistic settings [47,176,177,178,179,180,181,182,183]. These scalp-based methods provide complementary insights into the neural mechanisms underlying impairment and recovery and enable real-time monitoring of cortical activity. Compared to fMRI, fNIRS and EEG offer greater flexibility and ecological validity and are suitable for a wide range of clinical populations, making them ideal for longitudinal monitoring of neural activity in post-stroke aphasia and to support innovative clinical research and neurorehabilitation efforts [47,176,178,184,185,186,187,188].

#### 5.2.2. The Utility of EEG as a Biomarker of Impairment and Recovery in Aphasia

EEG records electrical activity generated by the brain. It measures voltage fluctuations from ionic current flows within neurons, particularly postsynaptic potentials of pyramidal cells within the neocortex [189,190]. Since EEG captures electrical activity, it serves as a direct measure of neural activity. The acquired EEG signal reflects the summation of synchronous neural activity across large populations of neurons, primarily arising from superficial cortical layers. While EEG has relatively low spatial resolution compared to techniques like fMRI, its exceptional temporal resolution makes it especially well-suited for studying the timing and dynamics of everyday language and cognitive processing [191]. EEG is sensitive to oscillatory activity within different frequency ranges related to a range of brain and cognitive states. For instance, increases in mid-frontal theta power (4–7 Hz) have been linked to cognitive control and conflict monitoring, while reductions in parietal alpha power (8–12 Hz) typically accompany increased attentional demands and task engagement [192,193]. EEG has been used extensively in both research and clinical contexts. Clinically, it has most commonly been used to diagnose epilepsy, monitor sleep disorders, and assess levels of consciousness. However, a growing body of research underscores its clinical utility in stroke rehabilitation [194,195,196] and, in particular, post-stroke aphasia [197,198,199,200,201,202,203,204].

Converging evidence from EEG studies of post-stroke aphasia has provided key insights into the potential applications of EEG for diagnosing impairments, monitoring recovery, and measuring treatment gains. The high temporal sensitivity of EEG is particularly valuable for identifying when, during the course of language processing, neural breakdowns occur—an insight critical for understanding mechanisms of aphasia recovery [199,205,206,207,208,209,210]. In cognitive neuroscience, EEG is often employed to examine event-related potentials (ERPs)—time-locked responses to specific stimuli or cognitive events—which can be used to isolate distinct components of sensory and cognitive processing [189]. Prior ERP work in aphasia has shown that the amplitude, latency, and scalp distribution of EEG signals can distinguish between different linguistic deficits. For instance, semantic impairments have been linked to abnormal ERP waveforms in earlier time windows, while phonological impairments are associated with abnormalities within later time windows, mirroring the typical time course of language processing in healthy individuals [211,212,213]. These ERP signatures not only reflect impairment profiles but also serve as potential biomarkers of spontaneous and treatment-induced recovery of post-stroke aphasia [197,214]. Beyond ERPs, time-frequency analyses of oscillatory activity have identified specific EEG frequency bands that index recovery potential in PWA. In particular, a number of studies have reported that greater delta power—especially in the ipsilesional hemisphere—is associated with greater impairment and poorer prognosis, whereas reductions in delta activity over time correspond to improved language function [206,207,209]. In contrast, a greater presence of alpha and beta oscillations has been linked to better recovery outcomes [200,215]. Collectively, prior EEG work in aphasia has demonstrated its clinical potential to detect distinct impairment types, track neuroplastic changes, and inform prognosis and treatment efficacy in aphasia rehabilitation.

While only a limited number of studies have used EEG to examine bilingual aphasia, three key investigations have helped to advance our understanding of bilingual aphasia. Khachatryan et al. [216] investigated cross-linguistic interference from L2 to L1 using a semantic association judgment task that incorporated interlingual homographs to systematically manipulate cross-language influences. Building on prior behavioral work (e.g., [9,217]), which found that cross-language priming effects vary as a function of pre-stroke proficiency and post-stroke impairment, this study utilized EEG to investigate the underlying neural mechanisms of these behavioral observations. The authors analyzed ERPs, focusing on differences between early (P200) and late (N400) ERP components, corresponding to lexical/phonological encoding and semantic processing, respectively. They found that ERP effects were highly individualized, varying both in timing and scalp distribution across BWA. Critically, they found that these distinct patterns aligned with participants’ reported bilingual language histories.

First, they found that BWA with greater L2 proficiency demonstrated greater L2 interference during L1 processing. BWA with lower reported pre- and post-stroke L2 exposure showed greater P200 modulations, interpreted as L2-based facilitation at the level of lexical access. Across participants, pre- and post-morbid L2 proficiency and exposure ratings were negatively correlated with P200 amplitude in high-interference conditions, suggesting that greater L2 proficiency was associated with more L2 influence during early L1 processing. Conversely, individuals with more extensive L2 experience demonstrated ERP effects in the N400 window, indicating cross-linguistic influence at the level of semantic processing. These results show that the impact of L2 on L1 processing in bilingual aphasia differs by processing stage—lexical versus semantic—and is shaped by individual language experience and exposure.

Importantly, despite comparable behavioral performance across patients, ERPs revealed nuanced, patient-specific neural profiles. This highlights the superior sensitivity of EEG in detecting subtle differences in language processing not captured by traditional assessments. Their findings support non-selective models of bilingual word recognition (e.g., BIA+, RHM), which posit that lexical access in one language activates related representations in both languages. Overall, this study demonstrates that EEG can detect distinct cross-language interference patterns in BWA that are modulated by pre- and post-morbid language experience, cognitive control, and impairment profiles. It underscores the clinical value of integrating electrophysiological data with detailed language history to better personalize assessment and intervention in bilingual aphasia.

Next, a case study by Radman et al. [218] investigated whether intensive lexical-phonological therapy provided in L2 would promote generalization to L1 for a Persian–French bilingual individual with Broca’s aphasia using both behavioral and ERP measures. The patient, a highly proficient L2 speaker, received four weeks of computer-assisted therapy targeting a set of French (L2) words. Behavioral assessment (BAT) and task-based EEG recordings were conducted in each language on separate testing days, both before and after treatment. The EEG tasks consisted of a picture naming task and a word-picture verification task. ERP analyses were performed on treated items only in both the treated (L2) and untreated (L1) languages.

Behavioral results indicated that the patient showed improved syntactic comprehension in both languages, as well as improvements in verbal comprehension, word and nonword repetition, and judgment in L1 only. For the picture naming task, improvements were restricted to the treated language (L2). The accompanying ERP findings were consistent with these behavioral observations, revealing significant post-treatment changes in ERP topography for treated L2 words only. Interestingly, the ERP changes were observed in a late (540–600 ms) time window associated with phonetic encoding [219,220]. These findings suggest that the therapy selectively modulated brain networks involved in phonological–phonetic processing in the treated language. The case study highlights the sensitivity of EEG in detecting targeted therapy effects and underscores its utility for tracking treatment-related neural changes and guiding individualized bilingual aphasia rehabilitation.

Finally, De Letter et al. [221] investigated the influence of bilingualism on the recovery of phonological processing post-stroke using EEG. Specifically, they examined ERP components across two timepoints in eleven bilingual and six monolingual individuals with aphasia using an auditory phoneme discrimination task. The authors found that mismatch negativity (MMN) latency and P300 amplitude were positive predictors of aphasia recovery. Interestingly, they found that BWA and MWA significantly differed in ERP characteristics, with BWA displaying reductions in MMN latency across time points, while MWA exhibited increases in MMN latency. The authors argued that BWAs are more likely than MWAs to improve in processing speed. They posited that bilinguals recruit domain-general cognitive control networks to support post-stroke language recovery, whereas monolinguals rely more on residual language circuitry. This pattern suggests that bilingualism may confer greater cognitive reserve in aphasia, potentially via enhanced engagement of control systems.

Taken together, these studies provide converging evidence that EEG is a powerful tool for advancing bilingual aphasia rehabilitation. The findings highlight that (1) both languages remain neurally active post-stroke, though to varying degrees [216]; (2) therapy in one language may lead to language-specific improvements, which can be captured by ERP components [218]; and (3) bilingualism may support better recovery of aphasia post-stroke, potentially due to greater cognitive and neural reserve [221]. Collectively, these studies emphasize the importance of individualized assessment and treatment strategies for BWA, and they illustrate the unique value of EEG in tracking neural markers of language processing and recovery in bilingual aphasia.

#### 5.2.3. Noninvasive Brain Stimulation: Overview and Rationale

Noninvasive brain stimulation (NIBS) refers to methods used to modulate neuronal excitability and plasticity through externally applied magnetic or electrical currents. Several of these methods, such as transcranial magnetic stimulation (TMS), transcranial direct current stimulation (tDCS), and transcranial alternating current stimulation (tACS), have shown considerable promise in post-stroke aphasia rehabilitation. In monolingual aphasia populations, NIBS protocols typically focus on either enhancing activity in left perilesional areas or inhibiting right hemisphere homologues, with their effectiveness varying based on individual patient characteristics such as lesion size and location, as well as residual activation patterns [46,222,223,224,225]. While NIBS has shown considerable promise for improving aphasia rehabilitation, its application to bilingual aphasia remains relatively underexplored. Nonetheless, emerging evidence suggests that NIBS may be a powerful tool for promoting language recovery in bilingual individuals by facilitating neural reorganization across both shared and language-specific networks [226].

A critical factor in the success of NIBS is its integration with behavioral language therapy. Evidence increasingly supports the idea that pairing neuromodulation with targeted linguistic tasks—either concurrently or sequentially—enhances treatment outcomes beyond what is achievable through behavioral therapy alone [223,224,227,228]. For example, a recent meta-analysis by Chai et al. [223] reported that combining NIBS with language therapy not only improved naming performance in people with aphasia but also yielded long-lasting treatment effects, suggesting that NIBS may help to promote long-term maintenance of language gains.

This combined approach is especially relevant for bilingual aphasia, where treatment protocols must account for the dynamics between the two languages. One strategy may involve tailoring therapy in both languages or selectively targeting the weaker language while applying stimulation to shared neural substrates to optimize CLG. A recent case study offers promising support for this rationale. Coemans et al. [229] investigated the effects of cerebellar tDCS combined with behavioral therapy in a French–Dutch bilingual with chronic aphasia. The patient received nine sessions of anodal tDCS to the right cerebellum, paired with therapy administered in L2 (Dutch). Compared to a sham condition, the active tDCS condition led to significant improvements in naming accuracy for both trained and untrained items in the treated language (within-language generalization), as well as in the untreated language (CLG). Gains also extended to untrained tasks, such as picture description and repetition, in both languages, suggesting that NIBS can also be successful in promoting transfer to broader language abilities.

Previous findings underscore the importance of selecting stimulation targets based not only on lesion location but also on their relevance to bilingual language control networks. While the left inferior frontal gyrus (IFG) remains a common target, the right hemisphere—particularly the right IFG—has emerged as an alternative site, especially in cases of extensive left hemisphere damage. Stimulation of the right IFG may modulate inhibitory control mechanisms that suppress left hemisphere activity, thereby supporting recovery [46]. Additionally, the cerebellum is gaining recognition as a novel target due to its contributions to both motor and language control. Together, these findings highlight the potential of NIBS—particularly when combined with behavioral therapy—to enhance language outcomes in bilingual aphasia and emphasize the need for individualized, neurologically-informed stimulation protocols that account for each patient’s language history and lesion profile.

## 6. Additional Cultural Considerations

The technological advances outlined in Section 5 hold tremendous promise for improving bilingual aphasia care, but their clinical impact depends critically on addressing cultural and equity considerations that have historically limited the field’s reach. Even the most sophisticated predictive models, adaptive therapies, and neurotechnologies will fail to serve diverse populations if they perpetuate assessment biases, ignore cultural communication patterns, or remain inaccessible to underserved communities. While Section 3 addressed the cultural validity challenges in assessment tools, this section focuses on broader cultural and equity considerations in treatment planning, service delivery, and research representation, identifying actionable strategies for addressing these challenges at both individual and systemic levels.

### 6.1. Culturally Responsive Treatment Planning

As discussed in Section 3 and Section 4, CLG mechanisms shape how gains in one language may benefit another [17]. In culturally responsive treatment planning, these mechanisms must be integrated with an understanding of the cultural contexts in which each language is used and valued. Goal selection should privilege participation in the communicative environments that matter most to the patient, reflecting the cultural and social roles that languages play in their daily life (e.g., [36,39,230]). This approach aligns treatment objectives with the International Classification of Functioning’s activity and participation domains [231], embedding patient-generated communication targets that reflect culturally meaningful activities.

Beyond individual goal-setting, culturally responsive treatment also requires attention to the broader social networks that sustain language use and cultural participation. A growing body of research suggests that social networks often shrink or shift after stroke, with documented declines in friendship ties, contact frequency, and overall network size compared to age-matched peers [232,233,234,235]. While most large-scale studies do not track the language associated with each social tie, case-based research shows that when a minority or less-used language lacks conversational partners, the language, and possibly its associated cultural roles, may gradually fall out of use [236]. Conceptual frameworks further highlight how the erosion of these language-specific ties can affect identity, intergenerational relationships, and access to culturally rooted practices such as religion or ceremony [39]. These findings support incorporating language-specific social network mapping into clinical intake procedures to support engagement in personally and culturally meaningful interactions post-stroke.

### 6.2. Service Delivery Infrastructure

Sustainable improvements in bilingual aphasia care depend on strengthening professional training. Only 8% of US speech-language pathologists identify as bi- or multilingual service providers [29], and training in working with bilingual populations remains limited. Further, few clinicians report receiving formal instruction in bilingual assessment or culturally responsive intervention in graduate school (e.g., [237]). This shortfall contributes to disparities in care and limits clinicians’ readiness to serve diverse populations. Workforce preparation could be strengthened by integrating bilingual aphasia modules into graduate curricula, expanding supervised multilingual practica in community settings, and embedding sociolinguistic competence as a core professional expectation, reflected in hiring, advancement, and ongoing professional development. These efforts should extend beyond language skills to include cultural humility, bias awareness, and collaborative practice with interpreters and cultural brokers.

Infrastructure gaps also hinder equitable service delivery. In many countries, interpreter access remains inconsistent [165,238,239,240]. When interpreters are involved, they should function as collaborative micro-teams rather than ad hoc supports. Recent work has identified specific strategies to support this collaborative model, including interpreter continuity and improved preparation practices [241]. Evidence from aphasia care in Indigenous communities further highlights the value of cultural brokers—trusted community members trained to bridge linguistic, cultural, and contextual gaps—working alongside clinicians through community-based programs, mobile clinics, or telepractice to address barriers related to distance, clinician monolingualism, and systemic under-resourcing [230]. AI-based assessment and therapy tools, as discussed in Section 5, may also help address service gaps, but they must be developed with sustained attention to cultural and linguistic equity to avoid reinforcing existing biases.

### 6.3. Research Representation and Evidence Base Diversity

A critical limitation underlying all cultural considerations is the evidence base itself. The bilingual aphasia literature remains concentrated in a limited number of language pairs, creating substantial representation gaps across global language communities [152,153]. This narrow focus limits the generalizability of findings and perpetuates inequities in evidence-based care for diverse populations [36,239,242].

While single-case reports in underrepresented languages demonstrate treatment feasibility (e.g., [236,243,244]), they cannot substitute for the systematic replication needed to establish clinical guidelines. A practical step toward addressing this gap could involve adopting standardized templates for reporting bilingual language history and for describing treatment procedures and outcomes in a consistent manner, as called for in recent work (e.g., [17,18,26]). Such standardization would allow single-case investigations across diverse language combinations to contribute meaningfully to meta-analytic reviews, building evidence systematically across diverse populations.

### 6.4. Moving Forward

Cultural factors are not peripheral to bilingual aphasia care but central to its effectiveness and equity. The evidence reviewed here reveals systematic disparities across assessment, treatment, technology, and research that limit the applicability of current approaches to diverse bilingual populations. Addressing these disparities requires both immediate clinical and technological adaptations and longer-term structural changes to ensure that scientific advances benefit all stroke survivors, regardless of their linguistic or cultural backgrounds.

## 7. Conclusions

The study of bilingual aphasia continues to challenge traditional models of language impairment and recovery, calling for frameworks that address the nuanced realities of bilingual communication. Encouragingly, developments in theory, research, clinical practice, and technology are beginning to converge, opening promising pathways for supporting recovery in bilingual individuals. Though significant questions remain, the field is steadily evolving toward more integrated, flexible approaches that honor the diversity of bilingual experience while aiming for care that is both individualized and ecologically valid.

## Data Availability

No new data were created or analyzed in this study. Data sharing is not applicable to this article.

## Abbreviations

The following abbreviations are used in this manuscript:

AI	Artificial Intelligence
AoA	Age of Acquisition
ASR	Automatic Speech Recognition
BAT	Bilingual Aphasia Test
BCSP	Bilingual Code-Switching Profile
BIA	Bilingual Interactive Activation
BLP	Bilingual Language Profile
BNT	Boston Naming Test
BSWQ	Bilingual Switching Questionnaire
BWA	Bilinguals with Aphasia
CAT	Comprehensive Aphasia Test
CLG	Cross-Language Generalization
EEG	Electroencephalography
ERP	Event-Related Potentials
fMRI	Functional Magnetic Resonance Imaging
fNIRS	Functional Near-Infrared Spectroscopy
IFG	Inferior Frontal Gyrus
L1	First-Acquired Language
L2	Second-Acquired Language
LEAP-Q	Language Experience and Proficiency Questionnaire
LHQ	Language History Questionnaire
LLM	Large Language Models
LSBQ	Language and Social Background Questionnaire
LUQ	Language Use Questionnaire
MMN	Mismatch Negativity
mSFA	Modified Semantic Feature Analysis
MWA	Monolinguals with Aphasia
NIBS	Noninvasive Brain Stimulation
PCA	Phonological Components Analysis
PPA	Primary Progressive Aphasia
PWA	People with Aphasia
RHM	Revised Hierarchical Model
RTSS	Rehabilitation Treatment Specification System
SFA	Semantic Feature Analysis
SHAP	SHapley Additive exPlanations
tACS	Transcranial Alternating Current Stimulation
tDCS	Transcranial Direct Current Stimulation
TLI	Treated Language Improvement
TMS	Transcranial Magnetic Stimulation
VNeST	Verb Network Strengthening Treatment
WAB	Western Aphasia Battery
WLG	Within-Language Generalization
